# Cross-continental environmental and genome-wide association study on children and adolescent anxiety and depression

**DOI:** 10.21203/rs.3.rs-2744140/v1

**Published:** 2023-04-18

**Authors:** Bishal Thapaliya, Bhaskar Ray, Britny Farahdel, Pranav Suresh, Ram Sapkota, Bharath Holla, Jayant Mahadevan, Jiayu Chen, Nilakshi Vaidya, Nora Perrone-Bizzozero, Vivek Benegal, Gunter Schumann, Vince D. Calhoun, Jingyu Liu

**Affiliations:** 1 Tri-Institutional Center for Translational Research in Neuro Imaging and Data Science; 2 Department of Computer Science, Georgia State University, Atlanta, USA; 3 School of Electrical and Computer Engineering, Georgia Institute of Technology, Atlanta, USA; 4 Centre for Population Neuroscience and Stratified Medicine, Department of Psychiatry and Neuroscience, Charité Universitätsmedizin Berlin, Germany; 5 Centre for Population Neuroscience and Precision Medicine, Institute for Science and Technology of Brain-inspired Intelligence, Fudan University, Shanghai, China; 6 Department of Integrative Medicine, National Institute of Mental Health and Neurosciences, Bangalore, India; 7 ADBS Neuroimaging Center, Department of Psychiatry, National Institute of Mental Health and Neurosciences, Bangalore, India; 8 Centre for Addiction Medicine, Department of Psychiatry, National Institute of Mental Health and Neurosciences, Bangalore, India; 9 Department of Neurosciences, University of New Mexico School of Medicine, Albuquerque, NM, USA

## Abstract

Anxiety and depression in children and adolescents warrant special attention as a public health issue given their devastating and long-term effects on development and mental health. Multiple factors, ranging from genetic vulnerabilities to environmental stressors, influence the risk for the disorders. This study investigated the impact of environmental factors and genomics on anxiety and depression in children and adolescents across three cohorts: the Adolescent Brain and Cognitive Development Study (US), the Consortium on Vulnerability to Externalizing Disorders and Addictions (India), and IMAGEN (Europe). Linear mixed-effect models, recursive feature elimination regression, and LASSO regression models were used to identify the environmental impact on anxiety/depression. Genome-wide association analyses were then performed for all three cohorts with consideration of significant environmental effects. The most significant and consistent environmental factors were early life stress and school risk. A novel SNP, rs79878474 in chr11p15, was identified as the most promising SNP associated with anxiety and depression. Gene set analysis found significant enrichment in regions of chr11p15 and chr3q26, in the function of potassium channels and insulin secretion, particularly Kv3, Kir-6.2, SUR potassium channels encoded by the KCNC1, KCNJ11, and ABCCC8 genes, respectively, in chr11p15. Tissue enrichment analysis showed significant enrichment in the small intestine and a trend of enrichment in the cerebellum. The study highlights the consistent impact of early life stress and school risk on anxiety and depression during development and suggests the potential role of mutations in potassium channels and the cerebellum region. Further investigation is needed to better understand these findings.

## Introduction

Anxiety and depression are now considered as two of the most frequent mental disorders that affect children and adolescents^1^. According to the United States Centers of Disease Control National Survey of Children’s Health, 7.1 % of children aged 3–17 years (about 4.4 million) have been diagnosed with anxiety, 3.2% have been diagnosed with depression (roughly 1.9 million)^2^, and this percentage increased to 11.7% for adolescents. The WHO has reported that one in every four children in India aged 13 to 15 suffers from depression. United Nations International Children’s Emergency Fund has reported that nine million adolescents in Europe (aged 10 to 19) are living with mental disorders, with anxiety and depression accounting for more than half of all cases (https://www.unicef.org/eu/stories/mental-health-burden-affecting-europes-children).

The causal pathways of anxiety and depression are not fully delineated yet, but the risk factors are multifaceted as shown in previous studies. Poverty^3,4^, dysfunctional family relationships and parental divorce^5,6^, child abuse^7,8^, and other stressful life events^9,10^ are well-known environmental risk factors for anxiety and depression. Furthermore, it has been discovered that teenagers who live in an area surrounded by trees and other green vegetation had a lower risk of severe depressive symptoms^11^. The impact of various levels of environmental factors from the individual level, to neighborhood and regional levels collectively in a broader setting across continents, has yet to be investigated to test the generalizability and specificity of environmental effects.

The largest genome-wide association study (GWAS) ever conducted for anxiety found substantial connections between self-reported anxiety and specific single nucleotide polymorphisms (SNPs) from a total of 200,000 participants^12^. Most of the identified risk SNPs are situated in non-coding areas, implying that these genetic variants may transmit the risk by regulating gene expression^13–16^. Depression has a genetic component as well, with heritability estimated 31% to 42% in twin studies of children and adolescents^17^. Several large GWAS recently conducted have provided top-risk SNPs to depression in general^18,19^. Additionally, substantial genetic correlations were observed between panic disorder, MDD, depressive symptoms, and neuroticism in a recent meta-analysis in European countries^20^. However, limited effort has been made to explicitly identify the genetic risks on adolescent depression and anxiety^21^, likely due to complicated genetic-environmental-developmental interactions. The current study is focused on understanding the genetic and environmental influence on anxiety and depression during development on a large geographic scale, with the hope to more clearly delineate the consistent genes and environmental effects across continents.

Our recent study^22^ used the Adolescent Brain and Cognitive Development Study (ABCD) data to identify genetic and environmental risk factors for anxiety and depression in children. We found that both environmental factors, such as early life stress (ELS), household income, and area crime, and genetic variants contribute to anxiety and depression in children. Environmental factors and genetics could explain 6.2% and 10–15% of the symptom severity variance, respectively. With mental health in children and adolescents being a pressing global issue, we extend the study to three cohorts across US, India, and Europe and explore the general and specific effects of different levels of environmental factors, and genetic risk to anxiety and depression in children and adolescents under diverse backgrounds.

## Methods

### Data and Participants

In this study, we analyzed data from three big cohorts: ABCD from US, IMAGEN from Europe, and the Consortium on Vulnerability to Externalizing Disorders and Addictions (c-VEDA) from India. Participants from each cohort all signed the consent form for the original studies, and the original studies were approved by local ethic committees.

#### The ABCD Dataset

ABCD is a large ongoing study following youths from age 9–10 into late adolescence^23^ to understand factors that increase the risk of physical and mental health problems. Participants were recruited from 21 sites across the US to represent various demographic variables. Data was obtained from ABCD Data Release 3.0 baseline assessments, including Parent-rated Child Behavior Checklist (CBCL), School Risk and Protective Factors Survey, Youth Family Environment Scale-Family Conflict, Longitudinal Parent Demographics Survey, Parent Neighborhood Safety/Crime Survey, Sum Scores Culture & Environment Youth, Residential History Derived Scores, and Youth Neighborhood Safety/Crime Survey. Of the total 11,875 baseline samples (ages 9– 10 years old), 8,513 were used after removing samples with missing values. More information about the study can be found at http://abcdstudy.org.

#### The cVEDA Dataset

The cVEDA is a cooperative initiative between the Medical Research Council, UK and the Indian Council for Medical Research on substance addiction and its link with mental illness^24^. Participants aged 6–23 were recruited from seven centers of five geographical regions of India^25^. We analyzed data from children (6–11) and adolescents (12–17) using questionnaires including Mini-International Neuropsychiatric Interview - KID (MINI-KID), Environmental Exposures Questionnaire, Adverse Childhood Experiences International Questionnaire, Indian Family Violence and Control Scale Questionnaire, Socioeconomic Status Questionnaire, and School Experience Questionnaire. More details about the questionnaires can be found in the Supplementary files. After removing missing values, we had data from 4,326 samples.

#### The IMAGEN Dataset

The IMAGEN consortium recruited over 2000 adolescents and their parents from eight centres in Europe and over four successive time points: baseline at age 14, follow-up 1 at age 16, follow-up 2 at age 19, and follow-up 3 at age 23, examining how biological, psychological, and environmental factors during adolescence may influence brain development and mental health^26^. More information about the study can be found at https://imagen-project.org/. Assessments at baseline from Life Events Questionnaire, Bully Questionnaire, and the Development and Well-Being Assessment Interview Questionnaire (DAWBA) were used in this study. More details about the questionnaires can be found in the Supplementary files. After removing the missing values, the total number of samples was 1,888.

### Defining environmental Factors

Based on the availability, we extracted environmental factors from the all three cohorts,including eight environmental factors (air pollution, population density, area crime, neighborhood safety, school risk, household income, family conflict, ELS) for the ABCD Cohort, five factors (air pollution, school risk, household income, family conflicts, ELS) for the cVEDA, and three factors (ELS, school risk, family conflicts) for the IMAGEN cohort. Each factor is derived from multiple variables assessing related issues, which were summed together to get a more general measure for that particular environmental factor. See Supplementary files for the details of the variables used.

### Defining the anxiety/depression score

One overall score was used to represent combined anxiety and depression severity due to the highly common occurrence: about 3 in 4 children with depression also had anxiety^27^. For the ABCD cohort, the parent-rated CBCL is used to determine the rate of depression/anxiety in children. CBCL is designed to detect emotional and behavioral problems in children and adolescents through 113 questions. We selected 13 variables from the CBCL to capture aspects of anxiety and depression. In cVEDA study, we used five variables in MINI-KID to identify the rate of Anxiety/Depression. In IMAGEN cohort, 62 variables in DAWBA were selected to measure anxiety/depression scores. For all three cohorts, the sum of these variables was used to measure the overall score of anxiety and depression. See the exact questions used in the Supplementary files.

### Genomic data preprocessing

ABCD provided imputed whole genome data in release 3.0, where imputation was performed using the TOPMed imputation server following the instruction at (https://topmedimpute.readthedocs.io/en/latest/prepare-your-data/). With the same approach, we performed the imputation for IMAGEN genomic data using the TOPMed Imputation Server^28^. Imputation of cVEDA genomic data was performed on the Michigan imputation server^29^ to have the South Asian Ancestry (SAS) reference panel, followed by LiftOver function to HG38 coordinates^30^. After imputation, further filtering steps were applied to SNPs including imputation confidence R2>0.3, genotyping missing rate < 0.05, minor allele frequency >0.01, and Hardy-Weinberg equilibrium threshold of 1e-06. Furthermore, the individuals with more than 3 standard deviations away from the samples’ heterozygosity rate mean were removed. Finally, we had 10908 subjects and 8812066 SNPs for ABCD, 1014 subjects and 4475075 SNPs for cVEDA, and 1831 subjects and 8785037 SNPs for IMAGEN respectively.

### Data Analyses

#### Data Harmonization with reference to ABCD Cohort

**Figure 1.**
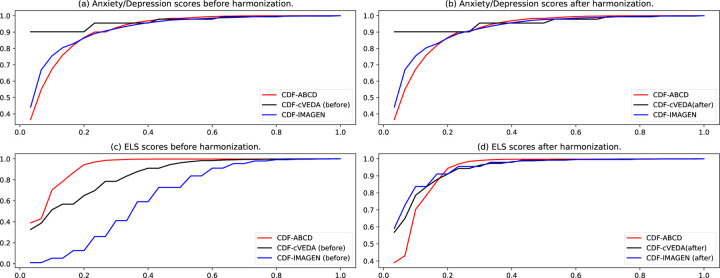
Data Harmonization. a) Anxiety/depression scores of ABCD, cVEDA and IMAGEN before applying gamma transformation. b) Anxiety/depression scores of ABCD, cVEDA and IMAGEN after applying gamma transformation (gamma=1.75x for cVEDA). No transformation needed for IMAGEN. c) ELS scores of ABCD, cVEDA and IMAGEN before applying gamma transformation. d) ELS scores of of ABCD, cVEDA and IMAGEN after applying gamma transformation (gamma=1.75x for cVEDA, 3.5x for IMAGEN)

The current study intends to assess the general effect of each environmental factor on children’s anxiety and depression across the three cohorts. As the utilized environmental and anxiety/depression assessments varied across the three cohorts, data harmonization is necessary to generate equivalent datasets from heterogeneous sources. Specifically, after scaling each factor into 0–1 range, we compared the cumulative distribution function (CDF) of each factor and appplied gamma transformation on cVEDA and IMAGEN factors while using ABCD factors as references. Gamma transformation defined as *y* = *X*^γ^ is a monotonic transformation where γ is chosen so that after transformation the CDF values at 90% of cVEDA and IMAGEN factors match that of ABCD factors. With this, we assume that each factor in the three cohorts has its own distribution (PDF), but 90% of samples fall into similar range. The selection of 90% is an empirical choice to align our data as shown in Figure 1, subject to change for different problems. The data harmonization was applied to anxiety/depression scores (cVEDA), ELS scores (cVEDA and IMAGEN), school risk scores(IMAGEN), air pollution scores(cVEDA), family conflict scores(cVEDA), and household income scores(cVEDA).

#### Analyzing effects of environmental factors using linear models

Environmental factors’ impact on anxiety/depression was studied using Linear Mixed Models (LMMs) for each factor, and Recursive Feature Elimination (RFE) and Least Absolute Shrinkage Selector Operator (LASSO) regression for factor combinations. For LMMs, each environment factor was tested separately in all cohorts with covariates of sex as fixed effect and site (and family only for ABCD) as random effects. Bonferroni corrections were applied for all tests. RFE and LASSO regression were used to identify the contributing environmental factors for anxiety/depression prediction. The dependent variable was anxiety/depression score, and the independent variables were all environmental factors (and sex) in each cohort. Cross-validation was applied for feature selection, and hold out test was used for effect size.

#### Genome wide association study (GWAS) for each cohort

A univariate LMM was used to test the genome-wide association through the software package: genome-wide efficient mixed-model association algorithm (GEMMA)^3^. The anxiety/depression score was the dependent variable and transformed with rank-based inverse normal transformation. We estimated the relatedness matrix based on SNPs using GEMMA to account for the relatedness between samples for all three cohorts. For ABCD cohort, covariates included the significant environmental factors identified in the previous tests along with the 10 eigenvectors of genomic SNP data that represent the population stratification and the relatedness matrix of ABCD samples (random effect). Similarly, for cVEDA cohort, covariates were the significant environmental factors along with age, the 10 genomic SNP eigenvectors and relatedness matrix of cVEDA samples (random effect). Covariates in IMAGEN cohort were the significant environmental factors, 10 eigenvectors and the relatedness matrix of IMAGEN samples (random effect). Merging the subjects with both the genetic data and the environmental factors available resulted in 7598 subjects for ABCD, 585 subjects for cVEDA, and 1580 subjects for IMAGEN respectively.

#### Meta analysis and Mega analysis

Both meta- and mega-analyses on genetic associations were performed to test the consistency of risk variants. We found 3,333,270 SNPs to be common across all three cohorts. For the meta-analysis, we applied the random effects model (RE2)^31^ from METASOFT on the results of individual GWAS performed for the three cohorts. RE2 model assumes different effect sizes across cohorts which are against a consist zero mean distribution under the null hypothesis. Mega-analysis was performed by combining all three cohorts’ data together and performing a genome-wide association analysis using GEMMA. The covariates included the relatedness matrix and the 10 eigenvectors computed from the combined genomic data, the common environmental factors that had consistent, significant effects across all three cohorts, as well as age and cohorts. Age was coded as two groups (1 for 6–11 age range, 2 for 12–17 age range), since the ABCD cohort has an age range of 9–10, cVEDA cohort has an age range of 6–11, and 12–17, and IMAGEN has 14.

#### Genomic risk loci and Gene mapping

Functional annotation was performed on SNP results from meta and mega-analyses with FUMA^32^, an online platform for the functional mapping of genetic variants. We defined ‘independent significant SNPs’ as those surpassing a predefined threshold P value (5E-06) and showing moderate to low linkage disequilibrium (r2 < 0.6), and defined ‘lead SNPs’ as the subset of independent SNPs (r2 < 0.1). FUMA identified genomic risk loci by merging LD blocks of independent significant SNPs that have close physical positions (< 250 kb). All LD information was calculated from the 1000G phase3 ALL population. Genes involved in each genomic risk loci were mapped from SNPs using three strategies in FUMA. More details can be found in FUMA website (https://fuma.ctglab.nl/tutorial)^32^.

#### Tissue specificity and gene set enrichment analyses

To explore if anxiety/depression associated mutations were enriched in specific human tissues, we performed tissue enrichment analysis for both meta-analysis and mega-analysis results by using MAGMA functions implemented in FUMA software. Briefly, gene expression data of different human tissues from the GTEx consortium were used to identify the genes that were differentially expressed in a specific tissue. Based on the individual SNPs association values, MAGMA quantifies the degree of association between a gene and anxiety/depression (i.e., obtain a gene-level P value) and tests if genes associated with anxiety/depression were enriched in the specifically expressed gene set in a specific tissue. More detailed information about tissue enrichment analysis can be found on FUMA website (https://fuma.ctglab.nl/).

The common genes mapped from both meta- and mega-analysis were selected to further investigate functional annotation using the GENE2FUNC function in FUMA. This function provides hypergeometric tests of enrichment in MSigDB gene sets^33^, including BioCarta, KEGG, Reactome, and Gene Oncology (GO). The P values for gene set enrichment analyses were adjusted by the Benjamini–Hochberg method. Furthermore, the common mapped genes were also tested for enrichment in specific human tissues by performing tissue enrichment analysis in FUMA^34^

## Results

### Significant effect of environmental factors on the anxiety/depression score

The data harmonization was performed on the anxiety/depression scores and some environmental factors of cVEDA and IMAGEN to match data from ABCD. As an illustration, Figure 1 shows the CDF of the anxiety/depression scores and ELS scores of the three cohorts before and after data harmonization. Other environmental factors’ CDF plots and parameters of gamma transformation can be in Supplementary files.

We analyzed harmonized data using three linear models to identify factors contributing to anxiety/depression scores in the three cohorts. The test results are listed in Table 1. In LMM models, ELS had the most significant effect on anxiety/depression scores in all three cohorts, with increasing ELS scores associated with higher anxiety/depression scores. School risk was also consistently significant, indicating that a better school environment was associated with lower anxiety/depression scores. Family conflict was significant in ABCD and cVEDA, but not IMAGEN. RFE and LASSO models to identify the contributing factors found that six environmental factors and sex explained 6.1% variance of the anxiety/depression score in the test data of ABCD cohort, three environmental factors and sex explained 9.1–9.9% variance in the cVEDA test data, and two environmental factors and sex explained 15–15.7% variance in the IMAGEN test data. These selected factors were used as covariates in the GWAS analysis of each cohort, respectively. The common significant contributors including ELS, school risk, and sex were used in the mega-analysis of GWAS in addition to age and cohort.

### Result of mega-analysis and meta-analysis on SNPs and genes

The genomic inflation factor (λ) in the QQ Plot for mega- and meta- analyses was 1.012 and 1.003 respectively, indicating no systemic bias in the analyses. Although, mega-analysis and meta-analysis did not find any SNPs to be significantly associated (p<5e-08) with anxiety/depression score, we found many promising SNPs with p values less than p<5e-06. The mega-analysis found 16 SNPs (Supplementary Table 5) to be promising with the most promising SNP as rs79878474 with p= 4.03e-07. The meta-analysis found 11 SNPs (Supplementary Table 4) to be promising with the same most promising SNP being rs79878474 (p=1.13E-06). In fact, the top three promising SNPs from mega-analysis and meta-analysis were the same (rs79878474, rs67861307, and rs6771812). The complete set of results of mega- and meta-analyses as well as each individual cohort’s analyses can be found in Supplementary Files.

We further used FUMA to identify independent risk loci in the promising SNPs from meta-analysis and mega-analysis, respectively. 7 independent risk loci were identified from mega-analysis (Table 2), mapped to 7 lead SNPs, 182 candidate SNPs, and 44 genes. Similarly, 7 independent risk loci were identified from the meta-analysis (Table 3), mapped to 7 lead SNPs, 82 candidate SNPs, and 58 genes. There are three common independent risk loci between meta- and mega- analyses: chr11:17545726, chr3:171071949, and chr6:38960253.

### Results of gene set and tissue enrichment analyses

20 common genes (Supplementary Table 13) from meta and mega-analysis were tested for the gene set enrichment. Among a total of 10,678 gene sets, 49 gene sets were statistically significant (Supplementary Table 10). They are grouped into three categories (positional, functional and GWAS Catalog) and consolidated with overlapped genes as listed in Table 4. The positional gene sets chr11p15 (p=8.35E-14) and chr3q36 (p=3.33E-07) had the lowest p-values. The GO biological processes gene sets include regulation of insulin/hormone/peptide secretion, and regulation of potassium channel. The GO cellular component gene sets include potassium channel complex, synapse, and axolemma. Reactome and KEGG databases identified similar related gene sets(Supplementary Table 10).

For the tissue enrichment analysis, when tested individually for meta- and mega-analysis results, both meta-and mega analysis results showed an elevated enrichment in the brain cerebellum with uncorrected p value of 0.007 and 0.003, respectively, tested for 53 tissue types (Supplementary Table 14 and 15), although not passing multiple comparison correction. In contrast, when performing the tissue enrichment test for 20 common genes, tissues in the small intestine showed significant enrichment with an adjusted p-value of 0.04. See Supplementary files for detailed results on tissue expression analysis using FUMA.

## Discussion

In this study, we examined the impact of environmental factors on anxiety and depression in children and adolescents across three diverse cohorts, and further studied genetic association with the consideration of environmental factors. Data harmonization allowed us to compare and analyze results from each cohort. The variance in anxiety and depression explained by environmental factors ranged from 6.1% to 15%. ELS and school risk were consistently selected by RFE and LASSO models with comparable effect sizes across cohorts, suggesting the effectiveness of data harmonization. It is noteworthy that school risk had a significant and consistent effect in addition to well known ELS impact, implying that improving school environments could help reduce anxiety/depression in youth. Family conflict, highly correlated to ELS, was not significant in the IMAGEN cohort and was eliminated by RFE and LASSO in ABCD and cVEDA due to not providing additional information. Family conflicts from IMAGEN presented very different CDF as compared to other cohorts; half of the participants in IMAGEN cohort reported family conflicts below 0.65, while half of the ABCD participants reported an incidence below 0.20 in a scale of 0 to 1. This could be due to more willingness to report the incidence of family conflicts in the case of IMAGEN, which might contribute to inconsistent effects.

Although mega- and meta- analyses both incorporate effects from three cohorts, mega-analysis assumes one homogeneous effect size from all three cohorts, while random-effect meta-analysis^31^ allows different effect size across cohorts. Our discussion mainly focuses on consistent findings from meta- and mega- analyses. Both analyses identified the same three top risk SNPs with the most promising SNP as rs79878474 with p value of 4.03E-7 (mega-analyses). This SNP is located in USH1C gene which is expressed in the brain (substantia nigra, hippocampus, putamen), following small intestine and spinal cord (C1–2) based on GTEx V8 (https://gtexportal.org/home/). Functionally, gene USH1C encodes a scaffold protein that functions in the assembly of Usher protein complexes and mutation of USH1C is known to be involved Usher syndrome type 1C and sensorineural deafness^35^. The other two top SNPs are in the TNIK gene, which is also highly expressed in brain and has been shown to regulate neurite development^36^, and mutations involved with an autosomal recessive form of cognitive disability^37^. But how these SNPs and genes related to anxiety/depression during development warrants further investigation.

FUMA identified three common independent risk loci with lead SNPs as rs79878474, rs6771812, rs6933332, and 20 common mapped genes between meta- and mega-analyses. The subsequent gene set analysis found 49 statistically significant gene sets. We want to highlight potassium channel regulation here with genes KCNJ11, KCNC1 and ABCC8 in chr11p15. Potassium (K+) channels locate in cell membranes and control the transportation of K+ ions efflux from and the influx into cells. This superfamily can be divided into many structural classes and located in different tissue types^38,39^. KCNC1 is primarily expressed in the cerebellum, almost exclusively in brain based on GTEx, and encodes member 1, subfamily C of integral membrane proteins that mediate the voltage-dependent potassium ion permeability of excitable membranes. This protein is the key to K+ voltage-dependent channel Kv3^39,40^. Kv3 channels regulate neurotransmitter release^41^, particularly affecting the high-frequency firing of neuron^42^. Alterations of Kv3 channels’ properties can cause severe neurological disorders like epilepsy and broad phenotypic spectrum including developmental delay^43^, schizophrenia^44^, and depression^45^. Recent animal and cell line studies have strengthened the connection between the Kv3 channel and depression. Mice with a reduced level of Kv3.1 presented vulnerability to depressive behavior, whereas up-regulation of Kv3.1 or acute activation of Kv3.1 induced resilience to depression^46^. A commonly used antidepressant drug, Fluoxetine, acts on Kv3 channels to affect Kv3.1b expression and serotonin secretion in a serotonergic cell line^47^, and another similar drug Vortioxetine inhibits delayed-rectifier K+ current caused by Kv3 channels activity in pituitary GH3 cells^48^. KCNJ11 is highly expressed in the cerebellum (the second highest besides muscle) and encodes an integral membrane protein that is the key to an inward-rectifier potassium channel, the Kir6.2 subunit of ATP- sensitive potassium channel. Kir6.2 channel is known to play an important role in modulating insulin secretion^39^, and also plays a role in stress adaptation^49,50^, as well as possibly part of the mechanism for anti-depression effect^50,51^. ABCC8 is also primarily expressed in cerebellum followed by the frontal cortex, pituitary, and pancreas. Functionally it modulates the SUR subunit of ATP-sensitive potassium channel which plays a key role in mediating glucose-stimulated insulin secretion. Recently new studies have linked insulin resistance with risk for depression and anxiety^52–54^. Our findings further strength this association by discovery of both potassium channel gene sets and insulin secretion gene sets in association of depression and anxiety score, and suggest that the Kv3, Kir 6.2,and SUR subunit of potassium channels may be important targets for anti-depression treatment.

The tissue enrichment analysis for either meta- or mega- analysis results showed an elevated expression in the cerebellum region of the brain. Common genes between meta- and mega-analyses showed significant tissue enrichment in the small intestine. The importance of cerebellum is supported by expression of key genes in the potassium channels as discussed above. Gene USH1C has the highest expression in small intestine, and genes INSC, SOX6, PLEKHA7, SLC2A2, and TNIK are all expressed in small intestine. The relation between small intestine and depression/anxiety has long been hinted particularly by brain-gut connections^55,56^. Our results emphasize small intestine and cerebellum in relation to depression and anxiety, which is not totally surprising but needs further in-depth investigation.

To summarize, our findings show that there is a consistent environmental influence, particularly ELS and school risks, on anxiety and depression in children and adolescents across continents. Further research into the genetic susceptibility highlights mutations in chromosome 11 p15 region, particularly potassium channels regulation gene set (Kv3, Kir 6.2, and SUR subunit) which are primarily expressed in the brain cerebellum. These findings, in line with literature about the potassium channel’s involvement in (anti)depression, and insulin secretion association with depression, motivate further investigation on how Kv3, Kir 6.2, SUR potassium channels in the cerebellum regulate anxiety and depression.

## Figures and Tables

**Table 1.** Output of RFE and LASSO models along with the individual environmental factors effect using Linear Mixed Models (LMMs).

ABCD Cohort					
Linear Mixed Models effect			RFE Model		
Environmental Factors	Effect Size(beta)	p-value	Selected Factors	Variance explained on training	Variance explained on testing
Early Life Stress (ELS)	0.355	1.31e-63	ELS, Household Income, Population Density, Area Crime, Neighborhood safety, School Risk, Sex	0.043	0.061
School Risk	−0.079	8.71e-26	**LASSO Model**		
Family Conflicts	0.046	1.50e-22	**Selected Factors**	**Variance explained on training**	**Variance explained on testing**
Neighborhood Safety	−0.086	1.09e-17	ELS, Household Income, Population Density, Area Crime, Neighborhood Safety, School Risk, Sex	0.039	0.061
Area Crime	−0.022	2.43e-06			
Household Income	−0.059	6.65e-06			
Air Pollution	−0.043	0.023			
Population Density	−0.027	0.031			
cVEDA Cohort					
Linear Mixed Models effect			RFE Model		
Environmental Factors	Effect Size (beta)	p-value	Selected Factors	Variance explained on training	Variance explained on testing
Early Life Stress (ELS)	0.424	1.62e-93	ELS, Household Income, School Risk, Sex	0.067	0.091
School Risk	−0.081	6.02e-06	**LASSO Model**		
Family Conflicts	0.063	1.40e-10	**Selected Factors**	**Variance explained on training**	**Variance explained on testing**
Household Income	−0.022	0.02	ELS, Household Income, School Risk, Sex	0.063	0.099
Air Pollution	−0.015	0.22			
IMAGEN Cohort					
Linear Mixed Models effect			RFE Model		
Environmental Factors	Effect Size (beta)	p-value	Selected Factors	Variance explained on training	Variance explained on testing
Early Life Stress (ELS)	0.304	2.80e-24	ELS, School Risk, Sex	0.142	0.157
School Risk	−0.182	1.73e-37	**LASSO Model**		
Family Conflicts	0.022	0.16	**Selected Factors**	**Variance explained on training**	**Variance explained on testing**
			ELS, School Risk, Sex	0.147	0.150

**Table 2.** Identification of independent loci from mega-analysis GWAS using FUMA

chr	LeadSNPPos	p	start	end	LeadSNPs
11	17545726	4.03E-07	17545726	17545726	rs79878474
3	171071949	1.03E-06	171066815	171073235	rs6771812
1	98433535	1.35E-06	98327133	98556159	rs11165937
12	131751769	1.82E-06	131729967	131775076	rs10744505
12	58377286	2.69E-06	58323136	58377286	rs11835606
3	5840111	2.99E-06	5831955	5849677	rs2437221
6	38960253	4.40E-06	38960253	38964657	rs6933332

**Table 3.** Identification of independent loci from meta-analysis GWAS using FUMA

chr	LeadSNPPos	p	start	end	LeadSNPs
11	17545726	1.13E-06	17545726	17545726	rs79878474
3	171071949	1.24E-06	171066876	171073235	rs6771812
2	38034558	1.25E-06	38031918	38034558	rs6755353
6	38960253	2.86E-06	38960253	38964657	rs6933332
3	80388728	3.49E-06	80388728	80493313	rs6764488
7	95705989	3.75E-06	95705989	95711226	rs756859
10	115522548	4.44E-06	115522548	115522548	rs2900993

**Table 4.** Identification of gene and gene sets associated with anxiety/depression using FUMA

	Significant Gene Sets	Adjusted p-value	Overlapped Genes
Positional Gene Sets	chr11p15	8.35E-14	CALCA:INSC:SOX6:PLEKHA7:NCR3LG1:KCNJ11:ABCC8:USH1C:MYOD1:KCNC1:SERGEF
	chr3q26	3.33E-07	EIF5A2:SLC2A2:TNIK:PLD1:GHSR
Functional Gene Sets	GO_AOLEMMA	8.59E-05	KCNJ11:KCNC1
	GO_POSITIVE_REGULATION_OF_CATION_CHANNEL_ACTIVITY	0.014	KCNJ11:ABCC8:KCNC1
	/GO_POTASSIUM_CHANNEL_COMPLEX	0.032	
	/REACTOME_POTASSIUM_CHANNELS	0.044	
	KEGG_TYPE_II_DIABETES_MELLITUS	0.002	KCNJ11:ABCC8:SLC2A2 / KCNJ11:ABCC8:SLC2A2:GLP1R:SERGEF:SLC2A2:GHSR:GLP1R
	/REACTOME_INTEGRATION_OF_ENERGY_METABOLISM	0.002	
	/GO_REGULATION_OF_INSULIN_SECRETION	0.002	
	/GO_REGULATION_OF_PEPTIDE_HORMONE_SECRETION	0.002	
	GO_NEGATIVE_REGULATION_OF_PEPTIDE_SECRETION	0.004	KCNJ11:ABCC8:SERGEF:GHSR/KCNJ11:ABCC8:SERGEF:SLC2A2:GHSR:GLP1R
	/GO_REGULATION_OF_PEPTIDE_SECRETION	2.73E-06	
	GO_SYNAPSE	0.032	CALCA:ABCC8:USH1C:KCNC1:TNIK:PLD1:GHSR
	GO_REGULATION_OF_SYSTEM_PROCESS	0.038	CALCA:KCNJ11:ABCC8:GHSR:GLP1R/CALCA:KCNJ11:ABCC8:GHSR:GLP1R:SOX6:MYOD1:KCNC
	/ GO_RESPONSE_TO_ENDOGENOUS_STIMULUS	0.018	
GWASCatalog Gene Sets	Body mass index	0.009	PLEKHA7:NCR3LG1:KCNJ11:ABCC8:USH1C:MYOD1:KCNC1:SERGEF
	Night sleep phenotypes	0.015	USH1C:MYOD1:KCNC1:SLC2A2:TNIK
	Systolic blood pressure x alcohol consumption interaction (2df test)	0.034	SOX6:PLEKHA7:KCNJ11
